# *t*-3-Ethyl-*r*-2,*c*-7-bis­(4-methoxy­phen­yl)-1,4-diazepan-5-one

**DOI:** 10.1107/S1600536809043311

**Published:** 2009-10-28

**Authors:** K. Ravichandran, P. Ramesh, S. Sethuvasan, S. Ponnuswamy, M. N. Ponnuswamy

**Affiliations:** a Centre of Advanced Study in Crystallography and Biophysics, University of Madras, Guindy Campus, Chennai 600 025, India; b Department of Chemistry, Government Arts College (Autonomous), Coimbatore 641 018, India

## Abstract

The title compound, C_21_H_26_N_2_O_3_, crystallizes with two independent mol­ecules in the asymmetric unit. In both independent mol­ecules, the diazepine ring adopts a chair conformation. In the crystal, the independent mol­ecules exist as N—H⋯O hydrogen-bonded *R*_2_^2^(8) dimers which are linked *via* N—H⋯O hydrogen bonds, forming tetra­mers. The tetra­mers are linked by C—H⋯O hydrogen bonds. In one of the molecules in the asymmetric unit, the terminal C atom of the ethyl group is disordered over two positions with refined occupancies of 0.742 (4) and 0.258 (4).

## Related literature

For general background to diazepine derivatives, see: Hirokawa *et al.* (1998 ▶); Jeyaraman & Ponnuswamy (1997 ▶); Senthil Kumar *et al.* (1992 ▶). For asymmetry parameters, see: Nardelli (1983 ▶). For puckering parameters, see: Cremer & Pople (1975 ▶). For hydrogen-bond motifs, see: Bernstein *et al.* (1995 ▶). For the synthesis, see: Jeyaraman *et al.* (1995 ▶); Ponnuswamy *et al.* (2006 ▶).
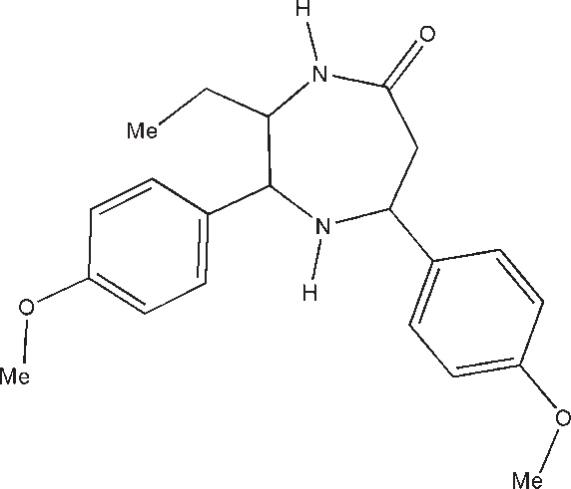


## Experimental

### 

#### Crystal data

C_21_H_26_N_2_O_3_
*M* *_r_* = 354.44
Triclinic, 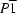

*a* = 10.5190 (3) Å
*b* = 13.3480 (4) Å
*c* = 15.0472 (4) Å
α = 102.118 (2)°
β = 93.662 (2)°
γ = 110.287 (2)°
*V* = 1915.89 (9) Å^3^
*Z* = 4
Mo *K*α radiation
μ = 0.08 mm^−1^
*T* = 293 K
0.25 × 0.23 × 0.20 mm

#### Data collection

Bruker Kappa APEXII area-detector diffractometer
Absorption correction: multi-scan (*SADABS*; Sheldrick, 2001 ▶) *T*_min_ = 0.980, *T*_max_ = 0.984
46036 measured reflections
10328 independent reflections
6341 reflections with *I* > 2σ(*I*)
*R*_int_ = 0.026

#### Refinement

*R*[*F* ^2^ > 2σ(*F* ^2^)] = 0.050
*wR*(*F* ^2^) = 0.149
*S* = 1.03
10328 reflections
495 parameters
H atoms treated by a mixture of independent and constrained refinement
Δρ_max_ = 0.21 e Å^−3^
Δρ_min_ = −0.18 e Å^−3^

### 

Data collection: *APEX2* (Bruker, 2004 ▶); cell refinement: *SAINT* (Bruker, 2004 ▶); data reduction: *SAINT*; program(s) used to solve structure: *SHELXS97* (Sheldrick, 2008 ▶); program(s) used to refine structure: *SHELXL97* (Sheldrick, 2008 ▶); molecular graphics: *ORTEP-3* (Farrugia, 1997 ▶); software used to prepare material for publication: *SHELXL97* and *PLATON* (Spek, 2009 ▶).

## Supplementary Material

Crystal structure: contains datablocks global, I. DOI: 10.1107/S1600536809043311/ci2933sup1.cif

Structure factors: contains datablocks I. DOI: 10.1107/S1600536809043311/ci2933Isup2.hkl

Additional supplementary materials:  crystallographic information; 3D view; checkCIF report

## Figures and Tables

**Table 1:** Hydrogen-bond geometry (Å, °)

*D*—H⋯*A*	*D*—H	H⋯*A*	*D*⋯*A*	*D*—H⋯*A*
N1*A*—H1*A*⋯O1*B*^i^	0.88 (2)	2.21 (2)	3.0833 (19)	172 (2)
N1*B*—H1*B*⋯O1*A*^i^	0.88 (2)	2.04 (2)	2.9179 (18)	175 (2)
N5*A*—H5*A*⋯O2*A*^ii^	0.91 (2)	2.49 (2)	3.3769 (18)	164 (2)
C19*B*—H19*B*⋯O3*B*^iii^	0.93	2.56	3.477 (2)	171
C20*A*—H20*A*⋯O3*B*^iv^	0.93	2.51	3.410 (2)	162
C20*B*—H20*B*⋯O1*B*^v^	0.93	2.53	3.398 (2)	156

Symmetry codes: (i) 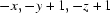; (ii) 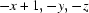; (iii) 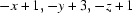; (iv) 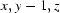; (v) 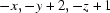.

**Fig. 1.**
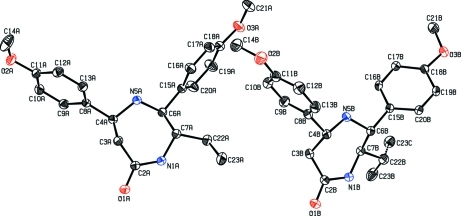
The asymmetric unit of the title compound. Displacement ellipsoids are drawn at the 30% probability level. H atoms have been omitted for clarity.

**Fig. 2.**
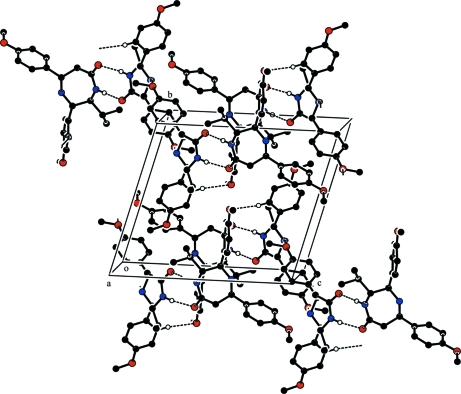
The crystal packing of the title compound, viewed along the a axis. H atoms not involved in hydrogen bonding (dashed lines) have been omitted for clarity.

## supplementary crystallographic information

### Comment

1,4-Diazepines are of considerable importance due to their wide spectrum of biological activities (Hirokawa *et al.*,  1998). Various substituted diazepan-5-ones have been synthesized using Schmidt rearrangement from the corresponding piperdin-4-ones and their stereochemistry has been reported (Senthil Kumar *et al.*, 1992; Jeyaraman & Ponnuswamy, 1997). In view of these importance and to ascertain the molecular conformation, a crystallographic study of the title compound, namely *t*-3-ethyl-*r*-2,*c*-7-bis(4-methoxyphenyl)-1,4-diazepan-5-one, was carried out.

In the title compound there are two crystallographically independent molecules in the asymmetric unit (Fig. 1). The diazepine ring in both molecules adopt chair conformation, with puckering (Cremer & Pople, 1975) and asymmetry (Nardelli, 1983) parameters q_2_ = 0.359 (2)Å, q_3_ = 0.702 (2)Å, φ_2_ = 132.2 (3)°, φ_3_ =102.1 (1)° and Δ_s_(N5A)= 0.017 (1)° for molecule A, and q_2_ = 0.378 (2) Å, q_3_ = 0.667 (2) Å, φ_2_ = -47.3 (3)°, φ_3_ =-75.9 (2)° and Δ_s_(N5B) = 0.022 (1)° for molecule B. The sum of bond angles around atoms N1A (359.9°) and N1B (359.6°) of the diazepine rings indicate *sp*^2^-hybridization, whereas the other N atoms [N5A (331.4°) and N5B (333.2°)] are *sp*^3^-hybridized.

In the crystal, independent molecules are linked by intermolecular N—H···O hydrogen bonds forming *R*_2_^2^(8) dimers. The adajacent centrosymmetric dimeric units are linked via N—H···O hydrogen bonds into a tetrameric unit with an *R*_2_^2^(16) ring motif (Bernstein *et al.* 1995). The tetramers are linked via C—H···O hydrogen bonds (Table 1).

### Experimental

In a typical reaction, t-3-ethyl-r-2,c-6-bis(4-methoxyphenyl)piperidin-4-one was first converted into its hydrochloride and then dry, powdered t-3-ethyl-r-2,c-6-bis(4-methoxyphenyl)piperidin-4-one hydrochloride (3.1 g) was added, in portions, to cold conc. H_2_SO_4_ (12.5 ml). The temperature of the solution was allowed to rise to 25°C and NaN_3_(0.75 g) was added in portions with vigorous stirring. The solution was poured into crushed ice and cold NaOH solution (2 N) was added slowly with stirring until the pH was 8. The separated white solid was filtered and crystallized using methanol (Jeyaraman *et al.*,  1995; Ponnuswamy *et al.*, 2006).

### Refinement

In one of the molecules in the asymmetric unit, the C atom of the ethyl group is disordered over two positions (C23B/C23C) with refined occupancies of 0.742 (4)and 0.258 (4). N-bound H atoms were located in a difference map and refined freely. C-bound H atoms were positioned geometrically (C-H = 0.93–0.98 Å) and allowed to ride on their parent atoms, with 1.5*U*_eq_(C) for methyl H and 1.2 *U*_eq_(C) for other H atoms.

### Crystal data

**Table:**

C_21_H_26_N_2_O_3_	*Z* = 4
*M**_r_* = 354.44	*F*(000) = 760
Triclinic, *P*1	*D*_x_ = 1.229 Mg m^−^^3^
Hall symbol: -P 1	Mo *K*α radiation, λ = 0.71073 Å
*a* = 10.5190 (3) Å	Cell parameters from 4523 reflections
*b* = 13.3480 (4) Å	θ = 1.4–29.2°
*c* = 15.0472 (4) Å	µ = 0.08 mm^−^^1^
α = 102.118 (2)°	*T* = 293 K
β = 93.662 (2)°	Block, colourless
γ = 110.287 (2)°	0.25 × 0.23 × 0.20 mm
*V* = 1915.89 (9) Å^3^	

### Data collection

**Table:**

Bruker Kappa APEXII area-detector diffractometer	10328 independent reflections
Radiation source: fine-focus sealed tube	6341 reflections with *I* > 2σ(*I*)
graphite	*R*_int_ = 0.026
ω and φ scans	θ_max_ = 29.2°, θ_min_ = 2.1°
Absorption correction: multi-scan (*SADABS*; Sheldrick, 2001)	*h* = −14→14
*T*_min_ = 0.980, *T*_max_ = 0.984	*k* = −17→18
46036 measured reflections	*l* = −20→20

### Refinement

**Table:**

Refinement on *F*^2^	Primary atom site location: structure-invariant direct methods
Least-squares matrix: full	Secondary atom site location: difference Fourier map
*R*[*F*^2^ > 2σ(*F*^2^)] = 0.050	Hydrogen site location: inferred from neighbouring sites
*wR*(*F*^2^) = 0.149	H atoms treated by a mixture of independent and constrained refinement
*S* = 1.03	*w* = 1/[σ^2^(*F*_o_^2^) + (0.06*P*)^2^ + 0.383*P*] where *P* = (*F*_o_^2^ + 2*F*_c_^2^)/3
10328 reflections	(Δ/σ)_max_ = 0.001
495 parameters	Δρ_max_ = 0.21 e Å^−^^3^
0 restraints	Δρ_min_ = −0.18 e Å^−^^3^

### Special details

**Table:**

Geometry. All e.s.d.'s (except the e.s.d. in the dihedral angle between two l.s. planes) are estimated using the full covariance matrix. The cell e.s.d.'s are taken into account individually in the estimation of e.s.d.'s in distances, angles and torsion angles; correlations between e.s.d.'s in cell parameters are only used when they are defined by crystal symmetry. An approximate (isotropic) treatment of cell e.s.d.'s is used for estimating e.s.d.'s involving l.s. planes.
Refinement. Refinement of *F*^2^ against ALL reflections. The weighted *R*-factor *wR* and goodness of fit *S* are based on *F*^2^, conventional *R*-factors *R* are based on *F*, with *F* set to zero for negative *F*^2^. The threshold expression of *F*^2^ > σ(*F*^2^) is used only for calculating *R*-factors(gt) *etc*. and is not relevant to the choice of reflections for refinement. *R*-factors based on *F*^2^ are statistically about twice as large as those based on *F*, and *R*- factors based on ALL data will be even larger.

### Fractional atomic coordinates and isotropic or equivalent isotropic displacement parameters (Å^2^)

**Table:**

	*x*	*y*	*z*	*U*_iso_*/*U*_eq_	Occ. (<1)
O1A	−0.01838 (15)	0.04649 (10)	0.27923 (10)	0.0830 (4)	
O2A	0.37857 (13)	−0.20475 (10)	−0.10577 (10)	0.0756 (4)	
O3A	0.69336 (15)	0.71516 (11)	0.19087 (10)	0.0811 (4)	
N1A	0.09646 (14)	0.22089 (11)	0.27464 (10)	0.0560 (3)	
H1A	0.0770 (18)	0.2418 (15)	0.3299 (13)	0.066 (5)*	
N5A	0.31252 (13)	0.21527 (10)	0.14281 (8)	0.0475 (3)	
H5A	0.3987 (18)	0.2259 (14)	0.1300 (12)	0.060 (5)*	
C2A	0.05133 (16)	0.11262 (14)	0.23888 (12)	0.0573 (4)	
C3A	0.09336 (15)	0.07227 (13)	0.14921 (12)	0.0549 (4)	
H3A	0.0620	0.1029	0.1031	0.066*	
H3B	0.0490	−0.0073	0.1296	0.066*	
C4A	0.24839 (15)	0.10354 (12)	0.15489 (10)	0.0462 (3)	
H4A	0.2850	0.1020	0.2159	0.055*	
C6A	0.32146 (15)	0.30438 (12)	0.22233 (10)	0.0475 (3)	
H6A	0.3576	0.2896	0.2778	0.057*	
C7A	0.17771 (16)	0.30792 (13)	0.23321 (11)	0.0515 (4)	
H7A	0.1292	0.2966	0.1720	0.062*	
C8A	0.28292 (14)	0.02316 (12)	0.08376 (10)	0.0443 (3)	
C9A	0.35352 (16)	−0.03896 (13)	0.10915 (12)	0.0533 (4)	
H9A	0.3810	−0.0303	0.1712	0.064*	
C10A	0.38390 (17)	−0.11330 (14)	0.04443 (13)	0.0595 (4)	
H10A	0.4327	−0.1534	0.0630	0.071*	
C11A	0.34280 (15)	−0.12864 (13)	−0.04710 (12)	0.0546 (4)	
C12A	0.27102 (17)	−0.06915 (15)	−0.07426 (12)	0.0604 (4)	
H12A	0.2418	−0.0795	−0.1363	0.072*	
C13A	0.24249 (17)	0.00635 (14)	−0.00872 (11)	0.0553 (4)	
H13A	0.1946	0.0470	−0.0276	0.066*	
C14A	0.3381 (3)	−0.2238 (2)	−0.20034 (18)	0.1147 (10)	
H14A	0.3690	−0.2786	−0.2335	0.172*	
H14B	0.3775	−0.1565	−0.2191	0.172*	
H14C	0.2399	−0.2494	−0.2132	0.172*	
C15A	0.42113 (16)	0.41145 (12)	0.20960 (10)	0.0486 (3)	
C16A	0.40863 (19)	0.44723 (14)	0.13156 (12)	0.0626 (4)	
H16A	0.3384	0.4028	0.0837	0.075*	
C17A	0.4976 (2)	0.54759 (15)	0.12202 (13)	0.0652 (5)	
H17A	0.4867	0.5698	0.0684	0.078*	
C18A	0.60159 (18)	0.61386 (13)	0.19175 (12)	0.0584 (4)	
C19A	0.6171 (2)	0.57884 (16)	0.26968 (14)	0.0773 (6)	
H19A	0.6882	0.6227	0.3171	0.093*	
C20A	0.52801 (19)	0.47928 (15)	0.27791 (12)	0.0677 (5)	
H20A	0.5402	0.4569	0.3313	0.081*	
C21A	0.6824 (3)	0.75565 (19)	0.11292 (18)	0.0952 (7)	
H21A	0.7529	0.8271	0.1218	0.143*	
H21B	0.5942	0.7613	0.1038	0.143*	
H21C	0.6926	0.7062	0.0599	0.143*	
C22A	0.18238 (19)	0.41711 (14)	0.29253 (14)	0.0676 (5)	
H22A	0.2236	0.4264	0.3547	0.081*	
H22B	0.2404	0.4768	0.2691	0.081*	
C23A	0.0429 (2)	0.42506 (18)	0.29508 (18)	0.0889 (7)	
H23A	0.0523	0.4952	0.3335	0.133*	
H23B	−0.0146	0.3671	0.3195	0.133*	
H23C	0.0024	0.4179	0.2340	0.133*	
O1B	−0.01626 (13)	0.72895 (10)	0.53207 (9)	0.0721 (4)	
O2B	0.10372 (17)	0.62330 (13)	−0.02416 (10)	0.0924 (5)	
O3B	0.65088 (12)	1.43287 (9)	0.47433 (8)	0.0594 (3)	
N1B	0.13486 (14)	0.90123 (11)	0.55429 (11)	0.0560 (3)	
H1B	0.1050 (19)	0.9184 (15)	0.6066 (13)	0.068 (5)*	
N5B	0.22122 (13)	0.94024 (10)	0.36776 (10)	0.0496 (3)	
H5B	0.2177 (18)	0.9690 (15)	0.3198 (13)	0.063 (5)*	
C2B	0.07613 (17)	0.79736 (13)	0.50562 (12)	0.0540 (4)	
C3B	0.12403 (18)	0.76579 (13)	0.41559 (12)	0.0576 (4)	
H3C	0.0724	0.6882	0.3878	0.069*	
H3D	0.2197	0.7752	0.4277	0.069*	
C4B	0.10920 (15)	0.83176 (12)	0.34669 (11)	0.0504 (4)	
H4B	0.0221	0.8429	0.3497	0.060*	
C6B	0.22491 (15)	1.02343 (12)	0.44994 (10)	0.0468 (3)	
H6B	0.1362	1.0323	0.4487	0.056*	
C7B	0.25490 (15)	0.98713 (12)	0.53712 (11)	0.0492 (3)	
H7B	0.3264	0.9563	0.5275	0.059*	
C8B	0.10992 (16)	0.77287 (12)	0.24925 (12)	0.0537 (4)	
C9B	0.1949 (2)	0.71538 (16)	0.22746 (13)	0.0682 (5)	
H9B	0.2532	0.7110	0.2744	0.082*	
C10B	0.1956 (2)	0.66371 (16)	0.13700 (14)	0.0719 (5)	
H10B	0.2527	0.6244	0.1239	0.086*	
C11B	0.1117 (2)	0.67125 (15)	0.06772 (13)	0.0680 (5)	
C12B	0.0291 (2)	0.72951 (17)	0.08731 (14)	0.0760 (5)	
H12B	−0.0265	0.7360	0.0399	0.091*	
C13B	0.02760 (19)	0.77921 (15)	0.17759 (13)	0.0663 (5)	
H13B	−0.0304	0.8178	0.1900	0.080*	
C14B	0.1884 (3)	0.5635 (2)	−0.04813 (17)	0.1003 (8)	
H14D	0.1733	0.5350	−0.1136	0.150*	
H14E	0.1670	0.5034	−0.0189	0.150*	
H14F	0.2826	0.6111	−0.0282	0.150*	
C15B	0.33542 (15)	1.13087 (12)	0.44774 (10)	0.0443 (3)	
C16B	0.46146 (15)	1.13398 (12)	0.42465 (11)	0.0494 (4)	
H16B	0.4742	1.0679	0.4039	0.059*	
C17B	0.56951 (15)	1.23233 (12)	0.43144 (11)	0.0492 (4)	
H17B	0.6533	1.2321	0.4153	0.059*	
C18B	0.55133 (15)	1.33051 (12)	0.46237 (10)	0.0459 (3)	
C19B	0.42406 (17)	1.32896 (13)	0.48211 (11)	0.0527 (4)	
H19B	0.4103	1.3949	0.5006	0.063*	
C20B	0.31804 (16)	1.23073 (12)	0.47456 (11)	0.0507 (4)	
H20B	0.2330	1.2311	0.4877	0.061*	
C21B	0.78088 (18)	1.44055 (16)	0.45013 (15)	0.0722 (5)	
H21D	0.8395	1.5167	0.4619	0.108*	
H21E	0.7710	1.4059	0.3860	0.108*	
H21F	0.8205	1.4042	0.4860	0.108*	
C22B	0.30606 (19)	1.08026 (14)	0.62487 (12)	0.0620 (4)	
H22C	0.3794	1.1421	0.6134	0.074*	0.742 (4)
H22D	0.2319	1.1048	0.6408	0.074*	0.742 (4)
H22E	0.2589	1.1293	0.6198	0.074*	0.258 (4)
H22F	0.2763	1.0489	0.6753	0.074*	0.258 (4)
C23B	0.3575 (3)	1.0464 (3)	0.70459 (18)	0.0822 (10)	0.742 (4)
H23D	0.3885	1.1077	0.7577	0.123*	0.742 (4)
H23E	0.4322	1.0234	0.6897	0.123*	0.742 (4)
H23F	0.2848	0.9864	0.7173	0.123*	0.742 (4)
C23C	0.4545 (7)	1.1483 (6)	0.6534 (5)	0.068 (2)	0.258 (4)
H23G	0.4667	1.2027	0.7096	0.102*	0.258 (4)
H23H	0.4889	1.1845	0.6063	0.102*	0.258 (4)
H23I	0.5035	1.1018	0.6628	0.102*	0.258 (4)

### Atomic displacement parameters (Å^2^)

**Table:**

	*U*^11^	*U*^22^	*U*^33^	*U*^12^	*U*^13^	*U*^23^
O1A	0.0985 (10)	0.0547 (8)	0.1012 (11)	0.0234 (7)	0.0645 (8)	0.0241 (7)
O2A	0.0739 (8)	0.0588 (8)	0.0864 (10)	0.0250 (6)	0.0276 (7)	−0.0034 (7)
O3A	0.0921 (9)	0.0530 (8)	0.0889 (10)	0.0080 (7)	0.0260 (7)	0.0266 (7)
N1A	0.0648 (8)	0.0478 (8)	0.0550 (9)	0.0184 (6)	0.0260 (7)	0.0107 (7)
N5A	0.0523 (7)	0.0408 (7)	0.0466 (7)	0.0133 (5)	0.0162 (5)	0.0089 (5)
C2A	0.0561 (9)	0.0494 (10)	0.0687 (11)	0.0183 (7)	0.0280 (8)	0.0160 (8)
C3A	0.0528 (8)	0.0437 (9)	0.0615 (10)	0.0118 (7)	0.0187 (7)	0.0063 (7)
C4A	0.0531 (8)	0.0423 (8)	0.0431 (8)	0.0159 (6)	0.0129 (6)	0.0119 (6)
C6A	0.0567 (8)	0.0420 (8)	0.0412 (8)	0.0152 (6)	0.0117 (6)	0.0084 (6)
C7A	0.0596 (9)	0.0434 (8)	0.0499 (9)	0.0169 (7)	0.0163 (7)	0.0095 (7)
C8A	0.0450 (7)	0.0387 (8)	0.0471 (8)	0.0111 (6)	0.0129 (6)	0.0120 (6)
C9A	0.0551 (8)	0.0558 (10)	0.0523 (9)	0.0220 (7)	0.0102 (7)	0.0174 (8)
C10A	0.0586 (9)	0.0529 (10)	0.0762 (12)	0.0275 (8)	0.0179 (8)	0.0205 (9)
C11A	0.0488 (8)	0.0416 (8)	0.0660 (11)	0.0111 (6)	0.0195 (7)	0.0042 (7)
C12A	0.0646 (10)	0.0664 (11)	0.0459 (9)	0.0230 (8)	0.0094 (7)	0.0068 (8)
C13A	0.0648 (9)	0.0577 (10)	0.0510 (10)	0.0296 (8)	0.0134 (7)	0.0159 (8)
C14A	0.1080 (18)	0.125 (2)	0.0847 (18)	0.0541 (16)	−0.0022 (13)	−0.0431 (15)
C15A	0.0585 (8)	0.0420 (8)	0.0428 (8)	0.0155 (7)	0.0142 (6)	0.0089 (6)
C16A	0.0763 (11)	0.0537 (10)	0.0449 (9)	0.0109 (8)	0.0049 (8)	0.0094 (8)
C17A	0.0883 (12)	0.0576 (11)	0.0512 (10)	0.0229 (9)	0.0196 (9)	0.0210 (8)
C18A	0.0675 (10)	0.0434 (9)	0.0622 (11)	0.0147 (8)	0.0231 (8)	0.0143 (8)
C19A	0.0807 (13)	0.0626 (12)	0.0640 (12)	−0.0022 (9)	−0.0042 (9)	0.0181 (10)
C20A	0.0776 (11)	0.0576 (11)	0.0542 (11)	0.0061 (9)	0.0004 (8)	0.0204 (9)
C21A	0.1160 (18)	0.0734 (15)	0.1049 (18)	0.0265 (13)	0.0362 (14)	0.0492 (14)
C22A	0.0770 (11)	0.0491 (10)	0.0749 (12)	0.0227 (9)	0.0280 (9)	0.0076 (9)
C23A	0.0942 (15)	0.0698 (13)	0.1167 (19)	0.0453 (12)	0.0381 (13)	0.0194 (13)
O1B	0.0800 (8)	0.0529 (7)	0.0832 (9)	0.0148 (6)	0.0376 (7)	0.0258 (6)
O2B	0.1108 (12)	0.0802 (10)	0.0665 (9)	0.0231 (9)	0.0147 (8)	−0.0024 (8)
O3B	0.0658 (7)	0.0392 (6)	0.0676 (7)	0.0129 (5)	0.0154 (5)	0.0112 (5)
N1B	0.0653 (8)	0.0476 (8)	0.0605 (9)	0.0214 (6)	0.0301 (7)	0.0177 (7)
N5B	0.0601 (7)	0.0386 (7)	0.0484 (8)	0.0145 (6)	0.0134 (6)	0.0120 (6)
C2B	0.0598 (9)	0.0456 (9)	0.0641 (10)	0.0216 (7)	0.0227 (8)	0.0215 (8)
C3B	0.0705 (10)	0.0409 (9)	0.0645 (11)	0.0209 (7)	0.0219 (8)	0.0152 (8)
C4B	0.0514 (8)	0.0403 (8)	0.0585 (10)	0.0154 (6)	0.0135 (7)	0.0113 (7)
C6B	0.0494 (8)	0.0399 (8)	0.0538 (9)	0.0192 (6)	0.0131 (6)	0.0109 (7)
C7B	0.0538 (8)	0.0450 (8)	0.0533 (9)	0.0208 (7)	0.0177 (7)	0.0145 (7)
C8B	0.0566 (9)	0.0382 (8)	0.0609 (10)	0.0109 (7)	0.0135 (7)	0.0106 (7)
C9B	0.0811 (12)	0.0688 (12)	0.0627 (12)	0.0358 (10)	0.0182 (9)	0.0166 (9)
C10B	0.0887 (13)	0.0601 (11)	0.0725 (13)	0.0326 (10)	0.0285 (10)	0.0140 (10)
C11B	0.0781 (12)	0.0481 (10)	0.0606 (12)	0.0072 (9)	0.0114 (9)	0.0046 (8)
C12B	0.0767 (12)	0.0727 (13)	0.0662 (13)	0.0204 (10)	−0.0025 (9)	0.0075 (10)
C13B	0.0665 (10)	0.0561 (11)	0.0683 (12)	0.0195 (8)	0.0042 (9)	0.0058 (9)
C14B	0.127 (2)	0.0764 (15)	0.0827 (16)	0.0288 (14)	0.0348 (14)	−0.0023 (12)
C15B	0.0538 (8)	0.0387 (8)	0.0440 (8)	0.0205 (6)	0.0127 (6)	0.0104 (6)
C16B	0.0554 (8)	0.0367 (8)	0.0592 (9)	0.0219 (6)	0.0152 (7)	0.0075 (7)
C17B	0.0510 (8)	0.0453 (9)	0.0531 (9)	0.0201 (7)	0.0137 (6)	0.0100 (7)
C18B	0.0585 (8)	0.0376 (8)	0.0404 (8)	0.0157 (6)	0.0089 (6)	0.0101 (6)
C19B	0.0702 (10)	0.0384 (8)	0.0578 (10)	0.0280 (7)	0.0199 (7)	0.0126 (7)
C20B	0.0579 (8)	0.0462 (9)	0.0578 (10)	0.0275 (7)	0.0209 (7)	0.0156 (7)
C21B	0.0618 (10)	0.0541 (11)	0.0878 (14)	0.0083 (8)	0.0136 (9)	0.0122 (10)
C22B	0.0760 (11)	0.0541 (10)	0.0546 (10)	0.0226 (8)	0.0163 (8)	0.0113 (8)
C23B	0.114 (2)	0.096 (2)	0.0519 (16)	0.0624 (19)	0.0119 (14)	0.0102 (14)
C23C	0.058 (4)	0.066 (5)	0.070 (5)	0.020 (3)	0.006 (3)	0.002 (4)

### Geometric parameters (Å, °)

**Table:**

O1A—C2A	1.2323 (19)	O3B—C21B	1.413 (2)
O2A—C11A	1.3725 (19)	N1B—C2B	1.328 (2)
O2A—C14A	1.402 (3)	N1B—C7B	1.4621 (19)
O3A—C18A	1.365 (2)	N1B—H1B	0.881 (19)
O3A—C21A	1.405 (3)	N5B—C6B	1.4667 (19)
N1A—C2A	1.330 (2)	N5B—C4B	1.4704 (19)
N1A—C7A	1.467 (2)	N5B—H5B	0.890 (19)
N1A—H1A	0.881 (19)	C2B—C3B	1.504 (2)
N5A—C4A	1.4642 (19)	C3B—C4B	1.527 (2)
N5A—C6A	1.4717 (19)	C3B—H3C	0.97
N5A—H5A	0.908 (17)	C3B—H3D	0.97
C2A—C3A	1.498 (2)	C4B—C8B	1.513 (2)
C3A—C4A	1.528 (2)	C4B—H4B	0.98
C3A—H3A	0.97	C6B—C15B	1.509 (2)
C3A—H3B	0.97	C6B—C7B	1.541 (2)
C4A—C8A	1.506 (2)	C6B—H6B	0.98
C4A—H4A	0.98	C7B—C22B	1.528 (2)
C6A—C15A	1.510 (2)	C7B—H7B	0.98
C6A—C7A	1.547 (2)	C8B—C13B	1.375 (2)
C6A—H6A	0.98	C8B—C9B	1.380 (2)
C7A—C22A	1.524 (2)	C9B—C10B	1.393 (3)
C7A—H7A	0.98	C9B—H9B	0.93
C8A—C13A	1.376 (2)	C10B—C11B	1.366 (3)
C8A—C9A	1.382 (2)	C10B—H10B	0.93
C9A—C10A	1.375 (2)	C11B—C12B	1.361 (3)
C9A—H9A	0.93	C12B—C13B	1.385 (3)
C10A—C11A	1.367 (3)	C12B—H12B	0.93
C10A—H10A	0.93	C13B—H13B	0.93
C11A—C12A	1.371 (2)	C14B—H14D	0.96
C12A—C13A	1.381 (2)	C14B—H14E	0.96
C12A—H12A	0.93	C14B—H14F	0.96
C13A—H13A	0.93	C15B—C16B	1.381 (2)
C14A—H14A	0.96	C15B—C20B	1.388 (2)
C14A—H14B	0.96	C16B—C17B	1.386 (2)
C14A—H14C	0.96	C16B—H16B	0.93
C15A—C16A	1.372 (2)	C17B—C18B	1.378 (2)
C15A—C20A	1.377 (2)	C17B—H17B	0.93
C16A—C17A	1.386 (2)	C18B—C19B	1.384 (2)
C16A—H16A	0.93	C19B—C20B	1.372 (2)
C17A—C18A	1.368 (3)	C19B—H19B	0.93
C17A—H17A	0.93	C20B—H20B	0.93
C18A—C19A	1.371 (3)	C21B—H21D	0.96
C19A—C20A	1.371 (2)	C21B—H21E	0.96
C19A—H19A	0.93	C21B—H21F	0.96
C20A—H20A	0.93	C22B—C23C	1.488 (7)
C21A—H21A	0.96	C22B—C23B	1.500 (3)
C21A—H21B	0.96	C22B—H22C	0.97
C21A—H21C	0.96	C22B—H22D	0.97
C22A—C23A	1.510 (3)	C22B—H22E	0.96
C22A—H22A	0.97	C22B—H22F	0.96
C22A—H22B	0.97	C23B—H22F	0.95
C23A—H23A	0.96	C23B—H23D	0.96
C23A—H23B	0.96	C23B—H23E	0.96
C23A—H23C	0.96	C23B—H23F	0.96
O1B—C2B	1.2341 (18)	C23C—H23G	0.96
O2B—C11B	1.380 (2)	C23C—H23H	0.96
O2B—C14B	1.405 (3)	C23C—H23I	0.96
O3B—C18B	1.3698 (18)		
			
C11A—O2A—C14A	117.77 (17)	C4B—C3B—H3C	108.6
C18A—O3A—C21A	118.48 (17)	C2B—C3B—H3D	108.6
C2A—N1A—C7A	127.11 (14)	C4B—C3B—H3D	108.6
C2A—N1A—H1A	115.7 (12)	H3C—C3B—H3D	107.6
C7A—N1A—H1A	117.1 (12)	N5B—C4B—C8B	107.49 (12)
C4A—N5A—C6A	115.07 (11)	N5B—C4B—C3B	111.39 (14)
C4A—N5A—H5A	109.4 (11)	C8B—C4B—C3B	112.06 (13)
C6A—N5A—H5A	107.9 (11)	N5B—C4B—H4B	108.6
O1A—C2A—N1A	121.74 (15)	C8B—C4B—H4B	108.6
O1A—C2A—C3A	120.31 (15)	C3B—C4B—H4B	108.6
N1A—C2A—C3A	117.89 (14)	N5B—C6B—C15B	107.71 (11)
C2A—C3A—C4A	112.81 (14)	N5B—C6B—C7B	110.00 (12)
C2A—C3A—H3A	109.0	C15B—C6B—C7B	110.86 (12)
C4A—C3A—H3A	109.0	N5B—C6B—H6B	109.4
C2A—C3A—H3B	109.0	C15B—C6B—H6B	109.4
C4A—C3A—H3B	109.0	C7B—C6B—H6B	109.4
H3A—C3A—H3B	107.8	N1B—C7B—C22B	107.11 (13)
N5A—C4A—C8A	109.97 (11)	N1B—C7B—C6B	112.21 (13)
N5A—C4A—C3A	110.15 (12)	C22B—C7B—C6B	114.44 (13)
C8A—C4A—C3A	111.13 (12)	N1B—C7B—H7B	107.6
N5A—C4A—H4A	108.5	C22B—C7B—H7B	107.6
C8A—C4A—H4A	108.5	C6B—C7B—H7B	107.6
C3A—C4A—H4A	108.5	C13B—C8B—C9B	117.20 (17)
N5A—C6A—C15A	108.36 (11)	C13B—C8B—C4B	119.98 (15)
N5A—C6A—C7A	110.41 (12)	C9B—C8B—C4B	122.77 (16)
C15A—C6A—C7A	112.49 (12)	C8B—C9B—C10B	121.68 (19)
N5A—C6A—H6A	108.5	C8B—C9B—H9B	119.2
C15A—C6A—H6A	108.5	C10B—C9B—H9B	119.2
C7A—C6A—H6A	108.5	C11B—C10B—C9B	119.39 (18)
N1A—C7A—C22A	106.99 (13)	C11B—C10B—H10B	120.3
N1A—C7A—C6A	111.92 (13)	C9B—C10B—H10B	120.3
C22A—C7A—C6A	113.26 (13)	C12B—C11B—C10B	120.02 (18)
N1A—C7A—H7A	108.2	C12B—C11B—O2B	115.65 (19)
C22A—C7A—H7A	108.2	C10B—C11B—O2B	124.33 (19)
C6A—C7A—H7A	108.2	C11B—C12B—C13B	120.16 (19)
C13A—C8A—C9A	117.36 (14)	C11B—C12B—H12B	119.9
C13A—C8A—C4A	121.57 (14)	C13B—C12B—H12B	119.9
C9A—C8A—C4A	121.05 (14)	C8B—C13B—C12B	121.54 (18)
C10A—C9A—C8A	121.20 (16)	C8B—C13B—H13B	119.2
C10A—C9A—H9A	119.4	C12B—C13B—H13B	119.2
C8A—C9A—H9A	119.4	O2B—C14B—H14D	109.5
C11A—C10A—C9A	120.46 (15)	O2B—C14B—H14E	109.5
C11A—C10A—H10A	119.8	H14D—C14B—H14E	109.5
C9A—C10A—H10A	119.8	O2B—C14B—H14F	109.5
C10A—C11A—C12A	119.60 (15)	H14D—C14B—H14F	109.5
C10A—C11A—O2A	115.66 (16)	H14E—C14B—H14F	109.5
C12A—C11A—O2A	124.74 (17)	C16B—C15B—C20B	117.40 (14)
C11A—C12A—C13A	119.48 (16)	C16B—C15B—C6B	120.88 (12)
C11A—C12A—H12A	120.3	C20B—C15B—C6B	121.58 (13)
C13A—C12A—H12A	120.3	C15B—C16B—C17B	122.12 (13)
C8A—C13A—C12A	121.89 (15)	C15B—C16B—H16B	118.9
C8A—C13A—H13A	119.1	C17B—C16B—H16B	118.9
C12A—C13A—H13A	119.1	C18B—C17B—C16B	119.22 (13)
O2A—C14A—H14A	109.5	C18B—C17B—H17B	120.4
O2A—C14A—H14B	109.5	C16B—C17B—H17B	120.4
H14A—C14A—H14B	109.5	O3B—C18B—C17B	124.78 (14)
O2A—C14A—H14C	109.5	O3B—C18B—C19B	115.76 (13)
H14A—C14A—H14C	109.5	C17B—C18B—C19B	119.45 (14)
H14B—C14A—H14C	109.5	C20B—C19B—C18B	120.45 (13)
C16A—C15A—C20A	116.87 (15)	C20B—C19B—H19B	119.8
C16A—C15A—C6A	122.38 (14)	C18B—C19B—H19B	119.8
C20A—C15A—C6A	120.73 (14)	C19B—C20B—C15B	121.22 (14)
C15A—C16A—C17A	121.91 (16)	C19B—C20B—H20B	119.4
C15A—C16A—H16A	119.0	C15B—C20B—H20B	119.4
C17A—C16A—H16A	119.0	O3B—C21B—H21D	109.5
C18A—C17A—C16A	119.78 (17)	O3B—C21B—H21E	109.5
C18A—C17A—H17A	120.1	H21D—C21B—H21E	109.5
C16A—C17A—H17A	120.1	O3B—C21B—H21F	109.5
O3A—C18A—C17A	124.94 (17)	H21D—C21B—H21F	109.5
O3A—C18A—C19A	115.86 (16)	H21E—C21B—H21F	109.5
C17A—C18A—C19A	119.20 (16)	C23C—C22B—C23B	71.7 (3)
C18A—C19A—C20A	120.19 (17)	C23C—C22B—C7B	121.4 (3)
C18A—C19A—H19A	119.9	C23B—C22B—C7B	112.83 (17)
C20A—C19A—H19A	119.9	C23C—C22B—H22C	37.5
C19A—C20A—C15A	122.03 (17)	C23B—C22B—H22C	109.0
C19A—C20A—H20A	119.0	C7B—C22B—H22C	109.0
C15A—C20A—H20A	119.0	C23C—C22B—H22D	124.9
O3A—C21A—H21A	109.5	C23B—C22B—H22D	109.0
O3A—C21A—H21B	109.5	C7B—C22B—H22D	109.0
H21A—C21A—H21B	109.5	H22C—C22B—H22D	107.8
O3A—C21A—H21C	109.5	C23C—C22B—H22E	107.6
H21A—C21A—H21C	109.5	C23B—C22B—H22E	133.3
H21B—C21A—H21C	109.5	C7B—C22B—H22E	106.7
C23A—C22A—C7A	113.26 (16)	H22C—C22B—H22E	79.3
C23A—C22A—H22A	108.9	H22D—C22B—H22E	31.7
C7A—C22A—H22A	108.9	C23C—C22B—H22F	105.9
C23A—C22A—H22B	108.9	C23B—C22B—H22F	38.0
C7A—C22A—H22B	108.9	C7B—C22B—H22F	107.6
H22A—C22A—H22B	107.7	H22C—C22B—H22F	139.1
C22A—C23A—H23A	109.5	H22D—C22B—H22F	76.2
C22A—C23A—H23B	109.5	H22E—C22B—H22F	106.8
H23A—C23A—H23B	109.5	C22B—C23B—H22F	38.5
C22A—C23A—H23C	109.5	C22B—C23B—H23D	109.5
H23A—C23A—H23C	109.5	H22F—C23B—H23D	105.4
H23B—C23A—H23C	109.5	C22B—C23B—H23E	109.5
C11B—O2B—C14B	117.88 (19)	H22F—C23B—H23E	140.1
C18B—O3B—C21B	118.75 (13)	H23D—C23B—H23E	109.5
C2B—N1B—C7B	127.36 (14)	C22B—C23B—H23F	109.5
C2B—N1B—H1B	116.5 (12)	H22F—C23B—H23F	75.6
C7B—N1B—H1B	115.7 (12)	H23D—C23B—H23F	109.5
C6B—N5B—C4B	118.50 (12)	H23E—C23B—H23F	109.5
C6B—N5B—H5B	107.2 (12)	C22B—C23C—H23G	109.5
C4B—N5B—H5B	107.5 (12)	C22B—C23C—H23H	109.5
O1B—C2B—N1B	121.57 (15)	H23G—C23C—H23H	109.5
O1B—C2B—C3B	120.71 (15)	C22B—C23C—H23I	109.5
N1B—C2B—C3B	117.70 (14)	H23G—C23C—H23I	109.5
C2B—C3B—C4B	114.62 (13)	H23H—C23C—H23I	109.5
C2B—C3B—H3C	108.6		
			
C7A—N1A—C2A—O1A	−178.83 (17)	C7B—N1B—C2B—C3B	−8.9 (3)
C7A—N1A—C2A—C3A	4.0 (3)	O1B—C2B—C3B—C4B	120.58 (17)
O1A—C2A—C3A—C4A	−115.07 (19)	N1B—C2B—C3B—C4B	−58.2 (2)
N1A—C2A—C3A—C4A	62.1 (2)	C6B—N5B—C4B—C8B	166.79 (13)
C6A—N5A—C4A—C8A	−162.77 (12)	C6B—N5B—C4B—C3B	−70.10 (17)
C6A—N5A—C4A—C3A	74.42 (16)	C2B—C3B—C4B—N5B	79.81 (17)
C2A—C3A—C4A—N5A	−84.26 (16)	C2B—C3B—C4B—C8B	−159.73 (14)
C2A—C3A—C4A—C8A	153.62 (14)	C4B—N5B—C6B—C15B	−171.56 (13)
C4A—N5A—C6A—C15A	165.11 (12)	C4B—N5B—C6B—C7B	67.51 (17)
C4A—N5A—C6A—C7A	−71.27 (16)	C2B—N1B—C7B—C22B	−164.28 (16)
C2A—N1A—C7A—C22A	170.41 (17)	C2B—N1B—C7B—C6B	69.3 (2)
C2A—N1A—C7A—C6A	−65.0 (2)	N5B—C6B—C7B—N1B	−76.30 (15)
N5A—C6A—C7A—N1A	77.41 (16)	C15B—C6B—C7B—N1B	164.68 (12)
C15A—C6A—C7A—N1A	−161.40 (13)	N5B—C6B—C7B—C22B	161.37 (13)
N5A—C6A—C7A—C22A	−161.54 (14)	C15B—C6B—C7B—C22B	42.35 (17)
C15A—C6A—C7A—C22A	−40.34 (19)	N5B—C4B—C8B—C13B	−93.95 (17)
N5A—C4A—C8A—C13A	−62.46 (18)	C3B—C4B—C8B—C13B	143.35 (16)
C3A—C4A—C8A—C13A	59.77 (19)	N5B—C4B—C8B—C9B	83.29 (19)
N5A—C4A—C8A—C9A	119.32 (15)	C3B—C4B—C8B—C9B	−39.4 (2)
C3A—C4A—C8A—C9A	−118.45 (16)	C13B—C8B—C9B—C10B	−1.4 (3)
C13A—C8A—C9A—C10A	1.0 (2)	C4B—C8B—C9B—C10B	−178.68 (16)
C4A—C8A—C9A—C10A	179.28 (14)	C8B—C9B—C10B—C11B	1.0 (3)
C8A—C9A—C10A—C11A	−1.0 (2)	C9B—C10B—C11B—C12B	0.5 (3)
C9A—C10A—C11A—C12A	0.1 (2)	C9B—C10B—C11B—O2B	−179.86 (17)
C9A—C10A—C11A—O2A	179.98 (14)	C14B—O2B—C11B—C12B	179.14 (19)
C14A—O2A—C11A—C10A	179.67 (18)	C14B—O2B—C11B—C10B	−0.5 (3)
C14A—O2A—C11A—C12A	−0.5 (3)	C10B—C11B—C12B—C13B	−1.5 (3)
C10A—C11A—C12A—C13A	0.7 (2)	O2B—C11B—C12B—C13B	178.86 (17)
O2A—C11A—C12A—C13A	−179.17 (15)	C9B—C8B—C13B—C12B	0.4 (3)
C9A—C8A—C13A—C12A	−0.2 (2)	C4B—C8B—C13B—C12B	177.77 (16)
C4A—C8A—C13A—C12A	−178.45 (14)	C11B—C12B—C13B—C8B	1.0 (3)
C11A—C12A—C13A—C8A	−0.7 (3)	N5B—C6B—C15B—C16B	−43.91 (19)
N5A—C6A—C15A—C16A	53.58 (19)	C7B—C6B—C15B—C16B	76.49 (17)
C7A—C6A—C15A—C16A	−68.77 (19)	N5B—C6B—C15B—C20B	140.46 (15)
N5A—C6A—C15A—C20A	−127.96 (17)	C7B—C6B—C15B—C20B	−99.15 (16)
C7A—C6A—C15A—C20A	109.69 (18)	C20B—C15B—C16B—C17B	2.8 (2)
C20A—C15A—C16A—C17A	−1.0 (3)	C6B—C15B—C16B—C17B	−173.01 (14)
C6A—C15A—C16A—C17A	177.51 (16)	C15B—C16B—C17B—C18B	0.1 (2)
C15A—C16A—C17A—C18A	0.1 (3)	C21B—O3B—C18B—C17B	2.8 (2)
C21A—O3A—C18A—C17A	−0.3 (3)	C21B—O3B—C18B—C19B	−176.19 (15)
C21A—O3A—C18A—C19A	−179.81 (19)	C16B—C17B—C18B—O3B	178.24 (14)
C16A—C17A—C18A—O3A	−178.51 (17)	C16B—C17B—C18B—C19B	−2.8 (2)
C16A—C17A—C18A—C19A	0.9 (3)	O3B—C18B—C19B—C20B	−178.41 (14)
O3A—C18A—C19A—C20A	178.55 (18)	C17B—C18B—C19B—C20B	2.6 (2)
C17A—C18A—C19A—C20A	−0.9 (3)	C18B—C19B—C20B—C15B	0.4 (2)
C18A—C19A—C20A—C15A	0.0 (3)	C16B—C15B—C20B—C19B	−3.1 (2)
C16A—C15A—C20A—C19A	1.0 (3)	C6B—C15B—C20B—C19B	172.70 (14)
C6A—C15A—C20A—C19A	−177.55 (18)	N1B—C7B—C22B—C23C	146.9 (4)
N1A—C7A—C22A—C23A	−62.2 (2)	C6B—C7B—C22B—C23C	−88.0 (4)
C6A—C7A—C22A—C23A	173.98 (16)	N1B—C7B—C22B—C23B	65.1 (2)
C7B—N1B—C2B—O1B	172.26 (16)	C6B—C7B—C22B—C23B	−169.84 (18)

### Hydrogen-bond geometry (Å, °)

**Table:**

*D*—H···*A*	*D*—H	H···*A*	*D*···*A*	*D*—H···*A*
N1A—H1A···O1B^i^	0.88 (2)	2.21 (2)	3.0833 (19)	172 (2)
N1B—H1B···O1A^i^	0.88 (2)	2.04 (2)	2.9179 (18)	175 (2)
N5A—H5A···O2A^ii^	0.91 (2)	2.49 (2)	3.3769 (18)	164 (2)
C19B—H19B···O3B^iii^	0.93	2.56	3.477 (2)	171
C20A—H20A···O3B^iv^	0.93	2.51	3.410 (2)	162
C20B—H20B···O1B^v^	0.93	2.53	3.398 (2)	156

Symmetry codes: (i) −*x*, −*y*+1, −*z*+1; (ii) −*x*+1, −*y*, −*z*; (iii) −*x*+1, −*y*+3, −*z*+1; (iv) *x*, *y*−1, *z*; (v) −*x*, −*y*+2, −*z*+1.
